# Bladder Cancer Cells Exert Pleiotropic Effects on Human Adipose-Derived Stem Cells

**DOI:** 10.3390/life12040549

**Published:** 2022-04-07

**Authors:** Małgorzata Maj, Łukasz Kaźmierski, Karolina Balik, Karolina Kowalska, Lidia Gackowska, Anna Bajek, Tomasz Drewa

**Affiliations:** 1 Department of Tissue Engineering, Chair of Urology and Andrology, Collegium Medicum, Nicolaus Copernicus University, Karłowicza 24, 85-092 Bydgoszcz, Poland; lukasz.kazmierski@cm.umk.pl (Ł.K.); karolinabalik@doktorant.umk.pl (K.B.); 294260@stud.umk.pl (K.K.); a.bajek@cm.umk.pl (A.B.); tomaszdrewa@cm.umk.pl (T.D.); 2 Department of Immunology, Collegium Medicum, Nicolaus Copernicus University, Sklodowskiej-Curie 9, 85-094 Bydgoszcz, Poland; l.gackowska@cm.umk.pl

**Keywords:** bladder cancer, adipose-derived stem cells, cell-based therapies, cancer recurrence

## Abstract

Stem cell-based therapies are considered one of the most promising disciplines in biomedicine. Bladder cancer patients could benefit from therapies directed to promote healing after invasive surgeries or to lessen urinary incontinence, a common side effect of both cancer itself and the treatment. However, the local delivery of cells producing large amounts of paracrine factors may alter interactions within the microenvironment. For this reason, reconstructive cellular therapies for patients with a history of cancer carry a potential risk of tumor reactivation. We used an indirect co-culture model to characterize the interplay between adipose-derived stem cells and bladder cancer cells. Incubation with ASCs increased MCP-1 secretion by bladder cancer cells (from 2.1-fold to 8.1-fold, depending on the cell line). Cancer cell-derived factors altered ASC morphology. Cells with atypical shapes and significantly enlarged volumes appeared within the monolayer. Incubation in a conditioned medium (CM) containing soluble mediators secreted by 5637 and HB-CLS-1 bladder cancer cell lines decreased ASC numbers by 47.5% and 45.7%. A significant increase in adhesion to ECM components, accompanied by reduced motility and sheet migration, was also observed after incubation in CM from 5637 and HB-CLS-1 cells. No differences were observed when ASCs were co-cultured with HT-1376 cells. Our previous and present results indicate that soluble mediators secreted by ASCs and bladder cancer cells induce opposite effects influencing cells that represent the non-muscle-invasive urinary bladder.

## 1. Introduction

Mesenchymal stromal cells (MSCs) represent a heterogeneous population of cells involved in tissue regeneration and homeostasis. They were initially identified in the bone marrow; however, further research has shown that they might be isolated from many fetal, neonatal, and adult tissues [1]. In an attempt to define the basic characteristics of human MSCs, the International Society for Cell and Gene Therapy (ISCT) Mesenchymal Stromal Cells (ISCT MSC) committee proposed minimal criteria for defining in vitro-expanded MSCs, i.e., (1) plastic adherence, (2) high expression of CD73, CD90, and CD105 and low expression of CD34, CD45, CD11b or CD14, CD19 or CD79α, and HLA-DR, (3) and multipotent differentiation potential [2].

MSCs migrate to the place of injury in response to chemotactic signals released in response to tissue damage. Activated by damage stimuli, MSCs secrete immunoregulatory mediators that modulate the immune response, promoting tissue repair. Additionally, MSCs regulate tissue regeneration by restoring the cellular components of the niche. They recruit functional cells to the niche, differentiate into missing cell components, and endow differentiated cells with stemness [3]. The protective effects of MSC-based therapies were initially ascribed to their local engraftment and subsequent differentiation. However, a growing body of evidence suggests that the observed effects are induced mainly by bioactive molecules secreted into the intercellular space [4].

MSC-based regenerative therapies involve isolation and subsequent transplantation of cells to damaged tissues. Adipose-derived stem cells (ASCs) are considered one of the most promising cell types for stem cell-based applications. They can be easily obtained using minimally invasive procedures and show potential for tissue repair [5]. ASC-based therapies have been successfully used for wound healing and soft tissue/bone regeneration [6]. They may also be considered regenerative treatments to promote healing for urological cancer patients. ASCs have already been used in early clinical trials for urinary incontinence treatment.

Nevertheless, reconstructive cellular therapies for patients with cancer histories may promote tumor growth [7]. Paracrine factors secreted by ASCs delivered near the tumor resection site may activate a rare subpopulation of persisting cancer stem cells (CSCs) that play an essential role in cancer relapse and progression. It has been shown that CSCs might also recruit and activate MSCs through paracrine mechanisms [8]. Thus, comprehensive knowledge of the crosstalk between tumor cells and MSCs seems essential to assess the potential risks of MSC-based regenerative therapies after cancer surgery.

Our previous results showed that bioactive molecules secreted by stem cells increased cancer cells’ viability and induced the activation of pro-survival signaling pathways [9]. As safety issues remain a significant limitation in translating MSC-based therapies into clinical practice here, we analyzed reverse interaction. Therefore, this study aimed to evaluate the influence of soluble mediators secreted by bladder cancer cells on proliferation, migration, immunophenotype, and other characteristics of ASCs that make them appealing in the field of regenerative medicine. 

## 2. Materials and Methods

### 2.1. Cell Lines

The 5637 and HT-1376 bladder carcinoma cell lines were obtained from the American Type Culture Collection (ATCC) and HB-CLS-1 cells from Cell Line Service (CLS). ASC52telo cells immortalized with hTERT were purchased from ATCC. They undergo quality control tests to guarantee cell identity. According to the International Society for Cell & Gene Therapy (ISCT) criteria, ASCs show high expression of several surface markers, including CD29, CD44, CD73, CD90, CD105, and CD166, and low expression of CD14, CD19, CD34, and CD45. When maintained under proper differentiation conditions, they retain a multipotent phenotype with the ability to differentiate into adipocytes, osteoblasts, and chondrocytes (verified at ATCC).

### 2.2. Cell Culture

All cell culture reagents and supplies, if not noted separately, were obtained from Corning. The 5637 and HB-CLS-1 cells were routinely cultured using RPMI1640, and HT-1376 cells were cultured in EMEM. Supplementation with 10% FBS was used for all media. ASC52telo cells were cultured in DMEM/Ham’s F-12 supplemented with 10% FBS and 10 ng/mL bFGF (Thermo Fisher Scientific, Waltham, MA, USA). Cells were maintained in a humidified atmosphere of 5% CO_2_ and 95% air at 37 °C. The medium was renewed every 2–3 days.

### 2.3. Co-Culture

ASC52telo cells were seeded in 96-well culture plates at a density of 5 × 10^3^ cells/cm^2^. At the same time, bladder cancer cells were seeded on cell culture inserts (PET 1 um) using the same cell number. Inserts were transferred to multi-well plates after a 24 h preincubation period. All experiments were performed after 72 h of cocultivation. ASCs maintained under standard conditions served as a control. Alternatively, they were cultured for 72 h in the secretome (conditioned medium) of cancer cells.

### 2.4. CM Preparation

Bladder cancer cells were seeded in standard TC-treated flasks at a density of 1 × 10^4^ cells/cm^2^. A conditioned medium (CM) containing metabolites, growth factors, and extracellular matrix proteins secreted by cancer cells was collected after 72 h of incubation. Samples were centrifuged, filtered (Steritop Membrane Filter, Merck Millipore, Burlington, MA, USA), and stored for later use at −20 °C. The CM was then thawed and mixed in a ratio of 1:1 with the growth medium used for ASC52telo cell culture. A medium from selected wells was exchanged for previously prepared CM to investigate the influence of bioactive molecules secreted by cancer cells on ASCs.

### 2.5. CM Composition

Bladder cancer cells were seeded in 6-well plates at a density of 5 × 10^3^ cells/cm^2^. Simultaneously, ASCs were seeded on polyethylene terephthalate (PET) hanging cell culture inserts (1 µm pore size). After a 24 h preincubation, the PET inserts were transferred to selected wells. The cells were co-cultured for 72 h without a medium change. The inserts were then removed, and the medium in all wells was exchanged. Secretomes obtained after 24 h of culture were used for further analysis. Bladder cancer cells from the monoculture served as a control. A Human Mix-N-Match Multi-Analyte ELISArray Kit (Qiagen) was used to profile the level of selected cytokines and chemokines. Antigen Standard Cocktail and experimental samples were transferred to a 96-well ELISA plate. After incubation and subsequent washing, the detection antibody was added. The excess of the antibody was removed during the wash step. Then, diluted Avidin-HRP was pipetted to all wells. After incubation and washing, the development solution was added. Finally, the stop solution was pipetted to each well in the same order as the development solution. A colorimetric reading from all wells was performed using a microplate reader (iMark, Bio-Rad, Tokyo, Japan) at 450 nm.

### 2.6. Cell Number

A Trypan Blue Staining Kit (Abcam) was used for cell counting in a Neubauer chamber. The trypan blue exclusion test is based on the fact that non-viable cells are permeable and take up the dye. After co-culture or culture in CM, cells were harvested and counted using a hemocytometer under a light microscope (Olympus).

### 2.7. Viability

An MTT Assay Kit (Abcam) was used to measure cell viability. The protocol is based on converting water-soluble thiazolyl blue tetrazolium bromide (MTT) to an insoluble formazan by viable cells. After co-culture or culture in CM, the growth medium was discarded, and the cells were incubated for 3 h at 37 °C with the MTT Reagent mixed with the same volume of serum-free media. After incubation, formazan crystals were dissolved in MTT Solvent. The absorbance was read at 590 nm on a microplate reader (iMark, Bio-Rad, Tokyo, Japan).

### 2.8. Proliferation

The BrdU Cell Proliferation ELISA Kit (Abcam) was used to measure cell proliferation. The protocol was based on incorporating BrdU (thymidine analog) into the DNA of actively proliferating cells. After culture in CM, the growth medium was discarded, and a fixing solution was added to each well. Cells were washed with Washing Buffer before incubation with Primary Detector Antibody and HRP Conjugate Antibody. After the final water wash, TMB solution was added to each well, and the plate was incubated in the dark. To stop color development, the stop solution was pipetted to all wells of the ELISA plate. The absorbance was read at 450nm on a microplate reader (iMark, Bio-Rad). Increasing ASC numbers was also measured during 72 h culture in CM from bladder cancer cells using live-cell imaging (JuLi Stage, NanoEntek, Seoul, Korea).

### 2.9. Exosome Release

Total Exosome Isolation Reagent (Thermo Fisher Scientific) was used to isolate exosomes released from ASCs cultured in CM. After 72 h of culture, the conditioned medium was replaced with a growth medium supplemented with 10% exosome-depleted FBS (Thermo Fisher Scientific). The medium was harvested after a subsequent 24 h culture, centrifuged, and mixed with Total Exosome Isolation Reagent. After overnight incubation, the samples were centrifuged at 1 × 10^4^× *g* for 1 h. Pelleted exosomes were resuspended in PBS. Protein concentration was measured with the µDrop Plate using a Mutliskan Sky microplate reader (Thermo Fisher Scientific).

### 2.10. Immunophenotype

A Human MSC Analysis Kit (BD Biosciences, San Jose, CA, USA) was used to analyze ASC immunophenotype. The kit includes a cell surface marker panel proposed by the ISCT for the minimal identification of human MSCs. After culture in CM, the cells were detached from the growth surface using accutase, washed, and suspended in stain buffer. ASCs at a concentration of 5 × 10^5^/mL were added to test tubes along with specific antibodies or positive/negative cocktails. After 30 min incubation, the cells were washed and suspended in Stain Buffer. Surface marker expression was analyzed via flow cytometry (FACS Canto II, BD Biosciences, San Jose, CA, USA).

### 2.11. ECM Adhesion

The ECM Cell Adhesion Array Kit (Merck) was used to compare cell adhesion to extracellular matrix (ECM) components. After culture in CM, the cells were harvested and suspended in Assay Buffer. ASCs were then transferred to wells pre-coated with a different ECM protein at a number of 5 × 10^4^ per well. After incubation, cells attached to the wells’ surfaces were washed and stained with Stain Solution. Extraction Buffer was added to solubilize cell-bound stain. The absorbance of the extract was read at 570 nm using a microplate reader (iMark, Bio-Rad).

### 2.12. Chemotaxis Cell Migration

The QCM Chemotaxis Cell Migration Assay (Merck) was used to study cell migration toward a chemoattractant-containing medium. The protocol utilizes a Boyden chamber, where the cells migrate through a semi-permeable membrane under different applied stimuli. After culture in CM, the cells were harvested and suspended in a chemoattractant-free medium. ASCs at a concentration of 1 × 10^5^/mL were added to inserts (8 µm pore size), and a medium containing 10% FBS was added to selected wells. After incubation, non-migratory cells were removed from the interior of the inserts using cotton-tipped swabs. The inserts were then transferred into unoccupied wells containing a dedicated Staining Solution. After staining and subsequent washing, the inserts were transferred to wells containing the Extraction Buffer to solubilize the cell-bound stain. The absorbance of the obtained extract was read at 570 nm using a microplate reader (iMark, Bio-Rad).

### 2.13. Wound Healing Assay

Collective cell migration was monitored using live-cell microscopy. Sheet migration is based on the movement of cell monolayers exposed to free space. After culture in CM, the monolayer was scratched with a pipette tip. The migration of either side of the gap was imagined for 24 h (until gap closure) using the live-cell imaging system JuLi Stage (Nanoentec, Seoul, Korea). The rate of gap closure, which is a measure of collective cell migration, was analyzed with Cellsens Dimension 3.1.1 (Olympus, Tokyo, Japan) using the custom-made artificial neural network. Images were acquired at 2-h intervals from all tested wells (from at least two sites per well) using a default brightfield protocol provided by JuLi Stage software. All wells of the multi-well plate unused during the experiment were filled with sterile, deionized water. The imaging system was enclosed inside a CO_2_ incubator for at least 24 h before the experiment to guarantee temperature equalization and minimize thermal drift. JPEG files from each well site were combined into time-lapse .tif files for neural network training and analysis. All image analyzes were performed on a Dell T5820 workstation equipped with an Nvidia Quadro P2200 video card.

### 2.14. Neural Network Training

Artificial neural networks (U-net based) were generated via a Deep Learning addon (demo version of the software provided via Olympus Poland) and were based on a ground truth consisting of at least 20 images. Images selected for the training were, by design, varied in quality, consisting of images with a consistent and non-ideal background to improve the final neural network quality. Three object classes were used for the training: cells, debris, and background. Objects in the training dataset were tagged manually by the tools included in the Deep Learning addon. The neural network training was finalized after at least 5 × 10^4^ iterations with at least 95% internal effectiveness (similarity of the analyzed images compared to the ground truth). The resulting neural network was compatible with the time-lapse .vsi files generated previously and validated by the end-user; if segmentation issues like false positive or false negative pixel classification were detected, new images were added to the training pool, and the neural network generation was repeated.

### 2.15. Neural Network Image Analysis

Artificial intelligence image analysis was performed entirely in Cellsens Dimension 3.1.1 using the custom-made neural network. All frames of the time-lapse files were analyzed, and the resulting main data consisted of an [%] of the total area covered by cells (object class–cells). The results were converted to .xlsx format for further analysis.

### 2.16. AKT/ERK Activation

An InstantOne ELISA Kit (Thermo Fisher Scientific) was used to measure phosphorylated ERK1/2, AKT 1/2/3, and p70 S6K from cell lysates. After culture in CM, the cells were harvested and suspended in HBSS buffer containing 5% FBS. ASCs were transferred to a 96-well ELISA plate at a number of 1 × 10^4^ per well and lysed with the use of a cell lysis mix reagent. The pre-made antibody cocktail was then added to selected wells. After an appropriate incubation time and multiple washing steps, the detection reagent was introduced to all wells. The absorbance of each well was read at 570 nm using a microplate reader (iMark, Bio-Rad) within 30 min of finalizing the assay with the stop reagent.

### 2.17. Statistical Analysis

The results are presented as means ± standard deviation (SD) of at least three independent experiments with three replicates per point. All analyzes were performed using STATISTICA 13.1 (StatSoft, Cracow, Poland). The Student’s *t*-test was used for two-group comparisons, and one-way ANOVA was used for comparing multiple groups. A confidence level of 95% was applied for all statistical tests.

## 3. Results

### 3.1. Multiplex Protein Analysis

The secretome composition was influenced by the environment to which th cancer cells were exposed (Table 1). Bioactive molecules secreted by ASCs increased monocyte chemoattractant protein-1 (MCP-1/CCL2) synthesis (from 2.1-fold to 8.1-fold, depending on the cell line). An increase in the concentration of IL-8 was also noted. No significant changes in other cytokines/chemokines levels were observed after co-culture with ASCs. IL-4, IFN-γ, and TNF-α levels were undetectable.

### 3.2. Cell Morphology

**Figure 1:**
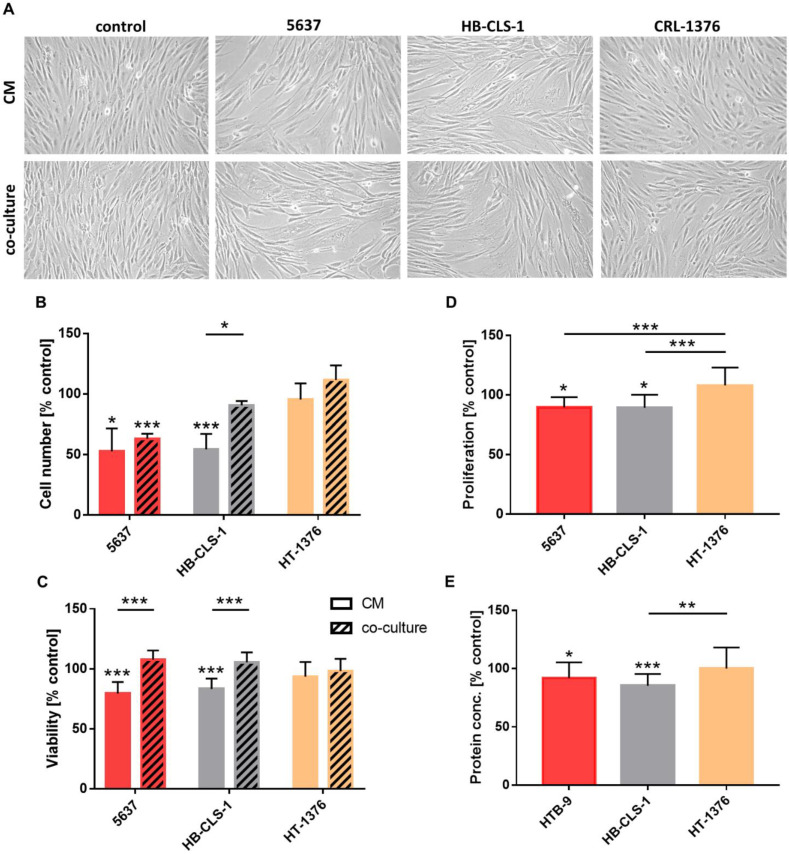
Growth characteristics of ASCs co-cultured with bladder cancer cells or in cancer cells CM. (**A**) Morphology of ASCs cultured in the presence of cancer cell-derived factors. Magnification x100. (**B**) ASC numbers decreased by 37% and 9.5% after co-culture with 5637 and HB-CLS-1 cells. Similarly, incubation in CM decreased the cell number by 47.5% and 45.7%. No significant differences were noted when ASCs were co-cultured with HT-1376 cells. (**C**) Conditioned medium from 5637 and HB-CLS-1 cells decreased ASC viability by 20.6% and 16.7%. No significant changes were observed after co-culture with bladder cancer cell lines. (**D**) Soluble mediators secreted by 5637 and HB-CLS-1 cells decreased the number of ASCs by 10.3% and 10.7% compared to the control. Co-culture with HT-1376 cells did not influence the proliferation of ASCs. (**E**) Culture in CM form 5637 and HB-CLS-1 cells reduced exosomal protein content by 8.1% and 17.7%. No differences were noted after culture in CM from HT-1376 cells. Red/orange stands for 5637 cells, gray for HB-CLS-1, and yellow for HT-1376 cells. All results are presented as a percentage of control. Bars represent standard deviation; * *p* > 0.05, ** *p* > 0.01, *** *p* > 0.001.

ASCs demonstrated morphology alternations after culture in the presence of soluble mediators secreted by bladder cancer cells (Figure 1A). Stem cells with atypical shapes and significantly enlarged volumes appeared within the monolayer. An increase in cellular size was not, however, accompanied by an increase in nuclear size. Microscopic images were used for quantitative analysis (cellSens Software, Olympus). No significant changes in nuclear volumes were detected in comparison to cultures in a standard growth medium (data not shown).

### 3.3. Cell Number

Trypan blue was used for cell counting. Co-culture with 5637 and HB-CLS-1 cells decreased ASC numbers by 37% (*p* < 0.001) and 9.5%, respectively, compared to the control (Figure 1B). Soluble mediators secreted by HT-1376 cells induced proliferation by 11.5%. These changes were not, however, statistically significant (*p* > 0.05). Incubation in the conditioned medium from 5637 and HB-CLS-1 CM decreased ASC numbers by 47.5% (*p* < 0.05) and 45.7% (*p* < 0.001), respectively, in comparison to the control. Interestingly, significant differences were noted between the proliferation of ASCs cultured in a conditioned medium and co-cultured with HB-CLS-1 cells.

### 3.4. Viability

The metabolic activity of ASCs decreased by 20.6% (*p* < 0.001) and 16.7% (*p* < 0.001) after culture in CM from 5637 and HB-CLS-1 cells, respectively (Figure 1C). No inhibiting effect of HT-1376 secretome on stem cell growth was observed. Interestingly, co-culture with bladder cancer cells did not affect the viability of ASCs. However, significant differences were observed between the metabolic activity of ASCs cultured in a conditioned medium and those co-cultured with 5637 and HB-CLS-1 cells.

### 3.5. Proliferation

Culture in CM from 5637 and HB-CLS1 cells decreased the proliferation rate of ASCs by 10.3% (*p* < 0.05) and 10.7% (*p* < 0.05), respectively, compared to the control (Figure 1D). No differences in BrdU incorporation into the newly synthesized DNA of actively proliferating cells were observed between those groups. Culture in CM from HT-1376 cells induced proliferation of ASCs by 8% in comparison to the control. These changes were not statistically significant (*p* > 0.05). Significant differences were observed between the cultures in CM from 5637 and HB-CLS-1 cells compared to HT-1376 cells.

### 3.6. Exosomal Protein Content

Soluble mediators secreted by 5637 and HB-CLS-1 cells reduced the total exosomal protein content (Figure 1E). Concentration of proteins in exosomes released by ASCs decreased by 8.1% (*p* < 0.05) and 17.7% compared to control (*p* < 0.01). No significant changes were noted after culture in CM from HT-1376 cells. Statistically significant differences were observed between cultures in CM from HB-CLS-1 cells compared to the HT-1376 cell line.

### 3.7. Immunophenotype

Simultaneous verification of several MSC-associated surface antigens increased confidence in the identification and validation of cultured cells. ASC52telo cells expressed positive markers characteristic of human MSCs (Supplementary Figure S1). No differences in the percentage of positive cells were observed after incubation in CM from bladder cancer cells (Table 2). Interestingly, the mean fluorescence intensity significantly increased for CD73 (from 32.2 to 46.2% depending on a cell line) and decreased for CD90 and CD105.

### 3.8. Migration and Adhesion to the ECM

**Figure 2:**
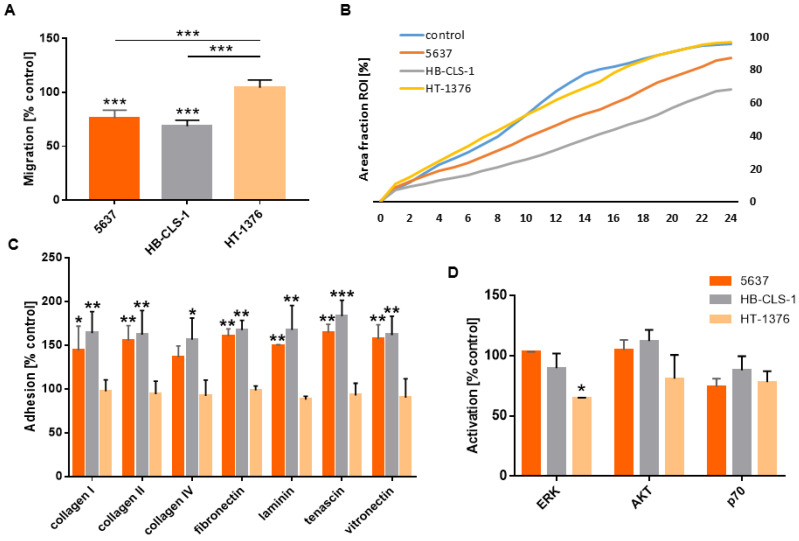
Adhesion and migration of ASCs cultured in CM from bladder cancer cells. (**A**) The migration of ASCs cultured in CM from 5637 and HB-CLS-1 cells decreased by 24.0% and 32.4%, respectively. No differences were observed for ASCs cultured in CM from HT-1376 cells. (**B**) Soluble mediators secreted by 5637 and HB-CLS-1 cells reduced sheet migration. After 24 h observation, 87.4% and 65.3% of the scratch wounds closed compared to 95.7% for cells cultured in a standard growth medium. No significant changes in growth kinetics were observed for ASCs cultured in CM from HT-1376 cells. (**C**) ASC adhesion significantly increased after culture in conditioned medium from bladder cancer cell lines. Depending on the ECM component, cell adhesion rose from 36.3% to 65.4% for ASCs cultured in CM from 5637 cells and from 56.1% to 83.0% for ASCs cultured in CM from HB-CLS-1 cells. (**D**) Culture with soluble mediators secreted by HT-1376 cells reduced ERK and AKT activation in ASCs by 37.4% and 18.8%, respectively. No differences were noted after culture in CM from 5637 cells. Although not statistically significant, decreased activation of P70 S6K was observed after culture in CM from all three bladder cancer cell lines. Red/orange stands for 5637 cells, gray for HB-CLS-1, and yellow for HT-1376 cells. All results are presented as a percentage of control. Bars represent standard deviation; * *p* > 0.05, ** *p* > 0.01, *** *p* > 0.001.

The cell migration assay was performed in a migration chamber based on the Boyden chamber principle. ASCs cultured in CM from 5637 and HB-CLS-1 cells reduced their motility by 24.0% (*p* < 0.001) and 32.4% (*p* < 0.001) in comparison to the control (Figure 2A). No significant changes in migration through the polycarbonate membrane were noted for ASCs cultured in CM from HT-1376 cells. Soluble mediators secreted by 5637 and HB-CLS-1 cells reduced the multi-cell migration by 8.6% (*p* < 0.01) and 27.4% (*p* < 0.01) (Figure 2B). There were also notable differences in the growth kinetics of cells cultured in CM from both cancer cell lines (Supplementary Figure S2 and Supplementary Video S1). No significant changes were noted for cells cultured in CM from HT-1376 cells. After 24 observations, gap closure (96.8%) was comparable to control (95.7%).

A significant increase in adhesion to all analyzed ECM components was observed after incubation in CM from the 5637 and HB-CLS-1 cells (Figure 2C). Adhesion to collagen I was increased by 44.1% (*p* < 0.05) and 64.3% (*p* < 0.01), to collagen II by 55.1% (*p* < 0.01) and 62.7% (*p* < 0.01), to collagen IV by 36.3% and 56.1% (*p* < 0.05), to fibronectin by 60.2% (*p* < 0.01) and 67.5% (*p* < 0.01), to laminin by 49.3% (*p* < 0.01) and 67.8% (*p* < 0.01), to tenascin by 65.4% (*p* < 0.01) and 83.0% (*p* < 0.001), and to vitronectin 57.6% (*p* < 0.01) and 62.4% (*p* < 0.01), respectively. No significant changes in adhesion to ECM components were noted for ASCs cultured in CM from HT-1376 cells.

### 3.9. AKT/ERK Activation

Expression of phosphorylated ERK1/2 decreased by 13.0% and 37.4% (*p* < 0.05) in ASCs cultured in CM from HB-CLS-1 and HT-1376 cells compared to the control (Figure 2D). Soluble mediators secreted by HT-1376 cells reduced AKT activation by 18.8%. P70 S6K phosphorylation decreased by 25.3%, 13.9%, and 21.5% after culture in CM from 5637, HB-CLS-1, and HT-1376 cells. However, these changes were not statistically significant.

## 4. Discussion

Adipose-derived stem cells are considered a suitable source of autologous material for cell-based reconstructive therapies after bladder cancer treatment [7]. For patients undergoing radical cystectomy with urinary diversion, residual urethra remains a common site of recurrence [10]. Numerous literature reports indicate the role of MSCs in different phases of carcinogenesis [11]. The fundamental role of ASC-induced epithelial-mesenchymal transition (EMT) in cancer progression has been recently highlighted [12,13]. On the contrary, several studies have reported their suppressive effects on cancer cells [14]. Hence, it seems that MSCs can both promote and restrain tumor growth [15,16,17]. Given that this bidirectional crosstalk is highly complex, we analyzed the influence of bladder cancer cells on the biological characteristics of stem cells derived from adipose tissue.

After co-culture with bladder cancer cells, ASCs with atypical shapes and significantly enlarged volumes appeared within the monolayer (Figure 1A). Al-Toub et al. reported that immortalized MSCs cultured in secretomes from head and neck cancer cells (FaDu) showed remarkable differences in shape. Similarly, MSCs incubated in CM from breast (MDA-MB-231), prostate (PC-3), and lung (NCI-H522) cancer cells did show changes in their appearance compared to control cultures [18]. We noticed that soluble mediators secreted by 5637 and HB-CLS-1 cancer cells significantly reduced ASC proliferation and viability (Figure 1B–D). Interestingly, cell number and viability decrease were more pronounced when cells were incubated in CM and not directly co-cultured with cancer cells. This may be due to differences in the composition of cancer cell-derived factors in monoculture and co-culture with ASCs. Paino et al. reported that MSCs cultured in CM from FaDu cells had a relatively slower growth rate than MSCs cultured in a standard growth medium. Changes in proliferation were accompanied by a decrease in G1 and an increase in the G2/M phase of the cell cycle [18]. Contrary to our results, breast cancer cells (MCF7) and osteosarcoma cells (SAOS2) induced ASC proliferation. Stem cells co-cultured with MCF7 and SAOS2 cells were distributed mainly in the S (38% and 17%) and G2/M phases (17% and 10%), while control ASCs in G0/G1 phase (92%) [19].

To investigate possible immunophenotype alternations, we analyzed the expression of stem cell markers. No statistically significant differences in CD44, CD73, CD90, or CD105 expression were noted (Figure 2). Paino et al. showed that CD90, CD29, and vimentin expression was similar for the control group and ASCs co-cultured with MCF7 and SASO2 cells. Nevertheless, a substantial variation in CD34 expression was observed. Further investigations confirmed that CD34 mRNA levels showed the same trend. MCF7 and SAOS2 cells induced upregulation of stemness markers (Sox2, Nanog, and OCT3/4). However, no differences in EMT-related genes (TWIST and Slug) were detected [19]. On the other hand, FACS analysis of MSCs separated after direct co-culture with cancer cells revealed the acquisition of epithelial cell-specific characteristics, including increased gene expression for cytokeratins and epithelial-like differentiation factors [20]. FaDu, MDA-MB-231, and PC-3, but not MCF7 cells, induced the expression of inflammatory cytokines and matrix metalloproteinases (MMPs). These results indicate that the effects of conditioned media from cancer cell lines on MSCs are cell line-dependent [18]. We also observed differences in the effects induced by bladder cancer cells on ASCs. 5637 and HB-CLS-1 cells strongly influenced the proliferation, viability, and migration of ASCs, while HT-1376 cells had no or little effect on their biological characteristics (Figure 1A–C). Genomic profiling revealed that HT-1376, unlike 5637 cells, was characterized with 10q deletion involving PTEN and no alteration of PIK3CA. TP53 inactivation was also detected [21]. These alternations induce invasive and metastatic potential of HT-1376 cells and may be responsible for observed cell-line-dependent differences. Similarly, only incubation in CM from 5637 and HB-CLS-1 cells reduced the total exosomal protein content (Figure 1E). Even though reports are limited, the exosome content seems to be altered when stem cells are cultured with cancer cells or in the tumor microenvironment [22].

Tumor-derived cell signaling molecules recruit MSCs to the tumor microenvironment [23]. We found that co-culture with ASCs promotes MCP-1 synthesis and secretion by bladder carcinoma cell lines (Table 1). Cancer cells produce MCP-1 to promote tumor growth and dissemination [24]. Grimm et al. reported that MCP-1 secreted by tumor-associated fibroblasts enhanced the migration and invasion of RT112 and Cal-29 bladder cancer cells [25]. The mechanisms underlying MCP-1-induced alterations in the tumor microenvironment remain unknown. Li et al. reported that MCP-1 induced the tumorigenesis of MCF-7 cells by suppressing E-cadherin, increasing MMP-2 activity, and stimulating migration/invasion. MCP-1 reduces GSK-3β activity through the MEK/ERK-mediated phosphorylation of serine-9 [26]. It has also been shown that MCP-1 induces osteosarcoma metastasis by MMP-9 overexpression [27]. MDA-MB-231 cells co-cultured with bone marrow MSCs enhanced de novo secretion of CCL5 (RANTES), which then induced cancer cell motility, invasion, and metastasis. MSCs migrated more avidly toward CM from cancer cells infused into the circulation of mice bearing MCF7 and MDA-MB-321 xenografts localized specifically to developing tumors [28]. MSCs provided a microenvironment for head and neck squamous cell carcinoma cell lines (HTB-43 and UM-SSC-22B) and induced their growth in a mouse in vivo model. Soluble mediators produced by cancer cells activated tumor-derived MSCs and increased secretion of IL-6, IL-8, (SDF)-1α and expression of CD54 marker [29].

Escobar et al. reported that MSCs cultured in CM from aggressive breast cancer cells (MDA-MB-231) secreted chemokines at higher levels than those cultured in the secretome of non-metastatic ER-positive MCF-7 cells [30]. Soluble mediators produced by metastatic cells induced MSC differentiation into cells resembling cancer-associated fibroblasts (CAFs), which showed sustained expression of SDF-1 and myofibroblast markers, including SMA and fibroblast surface protein [31]. Barcellos-de-Souza et al. identified TGF-β1 as a crucial molecule able to attract MSC to prostate carcinoma cells and tumor stroma components [32]. Similarly, it was demonstrated that tumor-derived osteopontin stimulated MSCs’ transformation into CAFs to promote tumor growth and metastasis [33]. Conditioned medium from breast cancer cells (4T1) promoted MSC motility by inducing cytoskeletal changes through activation of the RhoA pathway. MSCs exposed to CM showed abnormal phenotypes characterized by cell elongation, reduced F-actin stress fiber density, and adhesion [34]. In contrast, we showed that ASC adhesion to ECM components significantly increased after culture in CM from 5637 and HB-CLS-1 bladder cancer cell lines. Increased ECM adhesion was accompanied by reduced motility and sheet migration (Figure 2A–C).

Activation of numerous signaling cascades is required for MSC self-renewal. Gharibi et al. reported that PI3K/AKT and ERK pathways oppositely regulate MSC proliferation and differentiation to maintain stem cell homeostasis [35]. Hypoxic conditions induced bone marrow-MSCs proliferation and differentiation into endothelial cells via PI3K/AKT signaling. PI3K/AKT exerted anti-apoptotic effects by phosphorylating Bad and caspase-9. In addition, MSCs with increased AKT expression were more resistant to hypoxic-ischemic-like injury in vitro [36]. ERK activation was also required for VEGF-A/VEGFR2-induced differentiation of ASCs into endothelial cells [37]. Culture on vertically aligned silicon nanowire activated TGF-β/BMP, PI3K/AKT, MAPK, and Wnt pathways, which converged to stimulate MSC differentiation via the Ras-Raf-MEK-ERK cascade [38]. Li et al. reported that activation of ERK signaling was essential for umbilical cord-MSCs osteogenic differentiation [39]. ERK activation was also involved in the osteogenic differentiation of bone marrow-MSCs on surfaces modified by chemical functional groups [40]. Activated ERK phosphorylates various proteins that control cell proliferation, differentiation, apoptosis, migration, and metabolism. We showed that culture in CM from HB-CLS-1 and HT-1376 cells reduced the expression of phosphorylated ERK1/2 (Figure 2D). Therefore, reduced activation of intracellular signaling pathways may be responsible for observed alternations in ASC growth characteristics.

Considering the results of our present and previous studies, the possible triggering of cancer recurrence remains a significant concern in applying MSC-based therapies for bladder cancer patients, which may restrict their clinical use. In fact, despite tremendous effort to analyze the behavior of transplanted MSCs in vivo, long-term safety remains a significant limitation in translating stem cell therapies into clinical practice [41]. Another important factor contributing to the failure of MSC clinical development is inconsistent biological characterization. Depending on the tissue of origin, method of isolation, and in vitro propagation, MSCs differ in immunocompatibility, differentiation, and migratory capacity. Moreover, several studies have reported donor-related variations associated with gender, age, and accompanying diseases [42].

As the therapeutic effects of cell therapies are based, at least in part, on the secretion of EVs, they may be considered a novel MSC-based cell-free strategy with decreased risk [43]. MSC-derived EVs induce therapeutic effects comparable to MSCs as parental cells, and EVs contain equivalent content [44]. Moreover, it is relatively easy to modify the surface properties to enhance their therapeutic potential [42]. As EVs derived from bladder cancer patients contain miRNA amounts comparable to those isolated from non-oncogenic participants, they may be considered a cell-free alternative to MSCs [7]. Their potential for orchestrating tissue repair and regeneration without inducing oncogenic effects remains to be assessed. Another issue is the standardization of EV isolation, identification, and characterization. Knowing reproducible methods of EV production, macrovesicle-based therapies may experience rapid progress.

## 5. Conclusions

Adipose-derived stem cells also hold great potential for applications in cell-based reconstructive therapies for patients with cancer history. However, the risk of cancer progression associated with ASC transplantation remains unclear. We previously showed that ASCs increase bladder cancer cells’ proliferation, viability, and invasiveness through a pro-inflammatory phenotype. Here, we demonstrate that co-culture with ASCs promotes the secretion of MCP-1 by cell lines representing non-muscle-invasive urinary bladder cancer. Conditioned medium from 5637 and HB-CLS-1 cells reduced ASC metabolic activity and proliferation. An increase in adhesion to ECM components, accompanied by reduced motility and multi-cell migration, was also noted. Altogether, our findings indicate that soluble mediators secreted by ASCs and bladder cancer cells induce opposite effects influencing cells that represent non-muscle-invasive urinary bladder.

## Figures and Tables

**Table 1:** Changes in protein levels in the secretome of bladder cancer cells after co-culture with ASCs.

	5637		HB-CLS-1		HT-1376	
Protein	pg/mL	Fold Change	pg/mL	Fold Change	pg/mL	Fold Change
IL-1A	6.2 (1.90)	1.5	5.1 (2.32)	1.3	0.5 (0.08)	1.1
IL-1B	1.7 (0.30)	1.1	0.9 (0.03)	1.2	n.d.	n.d.
IL-6	35.5 (5.61)	1.6	15.4 (2.87)	1.4	n.d.	n.d.
IL-8	21.8 (4.02)	1.6	18.5 (4.92)	1.8	16.3 (3.72)	2.1 *
IL-10	n.d.	n.d.	n.d.	n.d.	0.6 (0.06)	1.7
GM-CSF	42.4 (6.36)	1.2	24.5 (5.43)	1.8	1.7 (0.43)	1.2
MCP-1	16.3 (3.45)	5.9 *	18.2 (1.98)	8.1 *	7.5 (1.56)	2.1 *
TGF-β1	13.9 (1.34)	1.1	18.6 (3.42)	1.1	12.6 (1.89)	1.2
RANTES	1.5 (0.22)	1.3	0.6 (0.05)	1.2	0.4 (0.02)	1.2

Standard deviation is given in brackets; n.d.–not detectable; * *p* < 0.05.

**Table 2:** Immunophenotype of ASCs cultured in CM from bladder cancer cells.

	CD44		CD73		CD90		CD105	
	Positive Cells [%]	IF Intensity	Positive Cells [%]	IF Intensity	Positive Cells [%]	IF Intensity	Positive Cell [%]	IF Intensity
5637	99.1 (0.3)	74.2 (15.7)	99.6 (0.4)	146.2 (8.4) *	97.4 (3.2)	69.9 (4.1)	94.9 (1.8)	49.0 (3.5) *
HB-CLS-1	99.0 (0.4)	80.9 (14.5)	99.2 (0.7)	144.8 (1.7) *	97.4 (2.8)	68.3 (6.1) *	94.2 (2.5)	46.0 (3.0) *
HT-1376	99.5 (0.3)	79.0 (7.8)	99.6 (0.3)	132.2 (4.1) *	97.8 (2.8)	67.5 (12.8) *	95.1 (2.2)	63.2 (2.3) *

Standard deviation is given in brackets. Immunofluorescence intensity is shown as the percentage of control. Standard deviation is given in brackets; * *p* < 0.05.

## Data Availability

The data presented in this study are openly available in the NCU repository at https://doi.org/10.18150/EZHF1T (accessed on 23 February 2022).

## Supplementary Materials

The following supporting information can be downloaded at: https://www.mdpi.com/article/10.3390/life12040549/s1, Figure S1: Representative flow cytometry histograms of surface marker expression on ASCs co-cultured with bladder cancer cells (out of 3 independent stainings); Figure S2: Representative images of sheet-migrating ASCs captured by the live-imaging system after incubation in CM from cancer cells. Green represents migrating cells and red cell debris; Video S1: Representative time-lapses of sheet-migrating ASCs captured by the live-imaging system after incubation in CM from cancer cells. Green represents migrating cells and red cell debris.
