# Supplementary material for: Bladder Cancer Cells Exert Pleiotropic Effects on Human Adipose-Derived Stem Cells
Source: Life (Basel). 2022 Apr 7;12(4):549. doi: 10.3390/life12040549 (PMC9025060; doi:10.3390/life12040549)
Supplement: Supplementary file 1 [file life-12-00549-s001.zip › supplementary Figures.pdf]

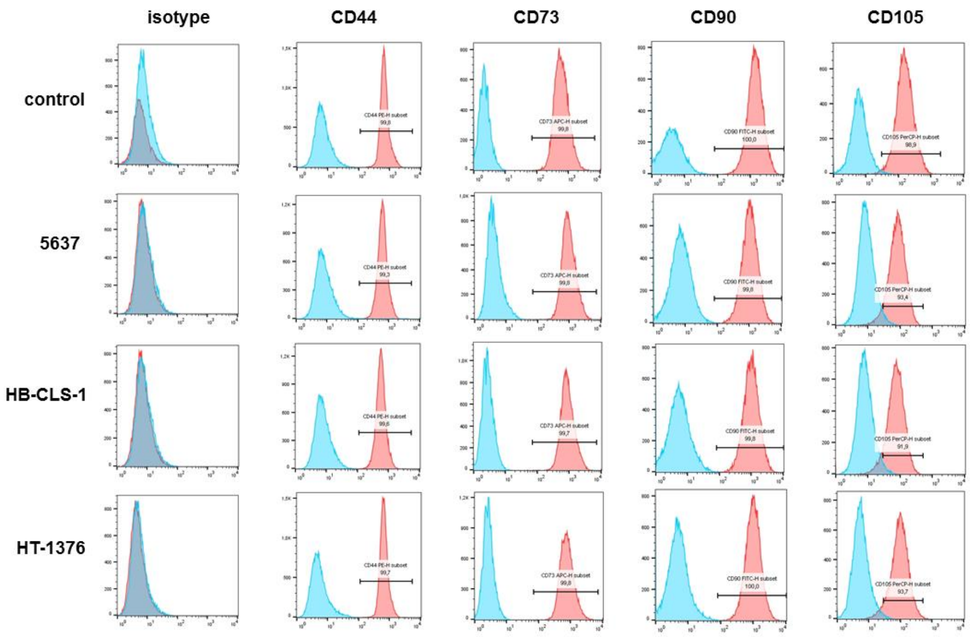

Figure S1: Representative flow cytometry histograms of surface marker expression on ASCs co-cultured with bladder cancer cells (out of 3 independent stainings).

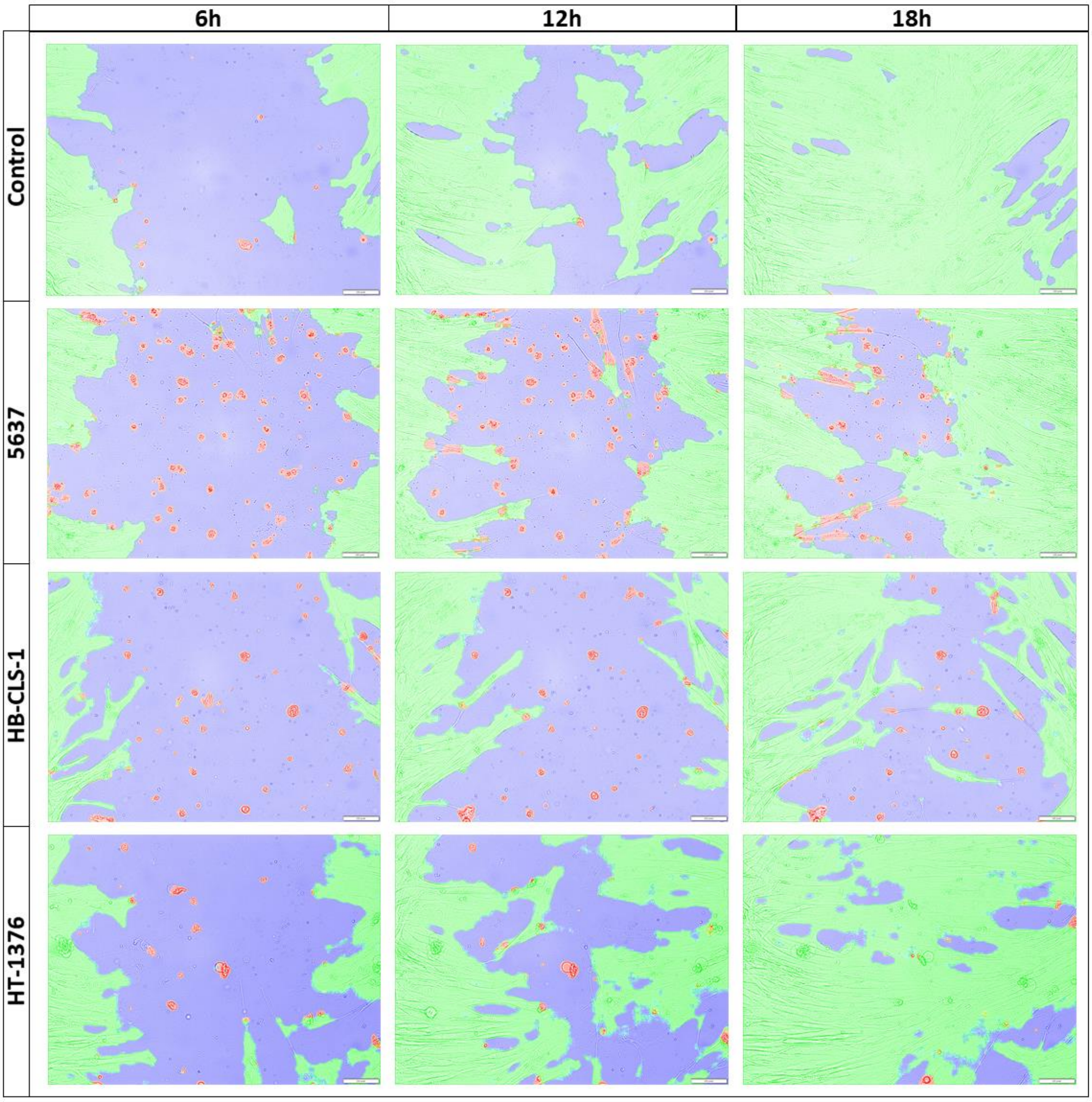

Figure S2: Representative images of sheet-migrating ASCs captured by the live-imaging system after incubation in CM from cancer cells. Green represents migrating cells and red cell debris.
